# 4-Meth­oxy-5-[4-(4-meth­oxy-1,3-benzodioxol-5-yl)perhydro-1*H*,3*H*-furo[3,4-*c*]furan-1-yl]-1,3-benzodioxole

**DOI:** 10.1107/S1600536808018138

**Published:** 2008-06-19

**Authors:** Rukmani Perumal Ezhilmuthu, Nagarajan Vembu, Nagarajan Sulochana

**Affiliations:** a Department of Pharmaceutics, Padmavathi College of Pharmacy, Dharmapuri 635 205, India; b Department of Chemistry, Urumu Dhanalakshmi College, Tiruchirappalli 620 019, India; c Department of Chemistry, National Institute of Technology, Tiruchirappalli 620 015, India

## Abstract

The 1,3-benzodioxole ring systems in the title compound, C_22_H_22_O_8_, are almost planar. The perhydro­furofuranyl system linking them adopts a distorted double-envelope conformation. Supra­molecular aggregation is effected by C—H⋯O, C—H⋯π and π–π [centroid–centroid distance of 3.755 Å, inter­planar distance of 3.633 Å and dihedral angle of 14.6°] inter­actions.

## Related literature

For related literature, see: Fu *et al.* (2006 ▶); Sonar *et al.* (2006 ▶); Hu *et al.* (2007 ▶); Zhou *et al.* (2007 ▶); Liang (2004 ▶); Wang *et al.* (2004 ▶); Zheng *et al.* (2005*a* ▶,*b* ▶); Hu *et al.* (2005 ▶); Qi *et al.* (2006 ▶); Hussain *et al.* (2006 ▶); Yu *et al.* (2006 ▶); Zhang *et al.* (2007 ▶); Betz *et al.* (2007 ▶); Yin *et al.* (2007 ▶); Beroza & Barthel (1957 ▶); Mitscher *et al.* (1979 ▶); Chien & Cheng (1970 ▶); Rao *et al.* (1981 ▶). For hydrogen bonds, see: Desiraju & Steiner (1999 ▶); Desiraju (1989 ▶). For graph-set notations, see: Bernstein *et al.* (1995 ▶); Etter (1990 ▶). For puckering parameters, see: Cremer & Pople (1975 ▶).
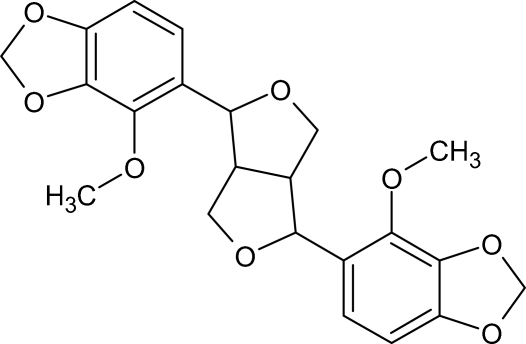


## Experimental

### 

#### Crystal data

C_22_H_22_O_8_
*M* *_r_* = 414.40
Monoclinic, 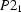

*a* = 4.754 (5) Å
*b* = 13.982 (4) Å
*c* = 14.672 (5) Å
β = 97.97 (6)°
*V* = 965.8 (10) Å^3^
*Z* = 2
Mo *K*α radiation
μ = 0.11 mm^−1^
*T* = 293 (2) K
0.3 × 0.3 × 0.3 mm

#### Data collection

Enraf–Nonius CAD-4 diffractometer
Absorption correction: ψ scan (North *et al.*, 1968 ▶) *T*_min_ = 0.805, *T*_max_ = 0.999
2000 measured reflections
1777 independent reflections
1505 reflections with *I* > 2σ(*I*)
*R*_int_ = 0.009
2 standard reflections every 100 reflections intensity decay: none

#### Refinement

*R*[*F* ^2^ > 2σ(*F* ^2^)] = 0.040
*wR*(*F* ^2^) = 0.110
*S* = 1.05
1777 reflections
272 parameters
1 restraint
H-atom parameters constrained
Δρ_max_ = 0.20 e Å^−3^
Δρ_min_ = −0.20 e Å^−3^

### 

Data collection: *CAD-4 Software* (Enraf–Nonius, 1994 ▶); cell refinement: *CAD-4 Software*; data reduction: *XCAD4* (Harms & Wocadlo,1995 ▶); program(s) used to solve structure: *SHELXS97* (Sheldrick, 2008 ▶); program(s) used to refine structure: *SHELXL97* (Sheldrick, 2008 ▶); molecular graphics: *PLATON* (Spek, 2003 ▶); software used to prepare material for publication: *SHELXL97*.

## Supplementary Material

Crystal structure: contains datablocks I, global. DOI: 10.1107/S1600536808018138/dn2355sup1.cif

Structure factors: contains datablocks I. DOI: 10.1107/S1600536808018138/dn2355Isup2.hkl

Additional supplementary materials:  crystallographic information; 3D view; checkCIF report

## Figures and Tables

**Table 1:** Selected torsion angles (°)

O2—C7—C8—C9	−13.3 (3)
C7—C8—C9—C10	−10.7 (3)
C11—C8—C9—C12	−9.0 (3)
C8—C9—C10—O2	32.0 (3)
C9—C8—C11—O1	−11.5 (3)
C8—C9—C12—O1	26.4 (3)

**Table 2:** Hydrogen-bond geometry (Å, °) *Cg*5 is the centroid of the C1–C6 ring and *Cg*6 is the centroid of the C13—C18 ring.

*D*—H⋯*A*	*D*—H	H⋯*A*	*D*⋯*A*	*D*—H⋯*A*
C5—H5⋯O2	0.93	2.34	2.721 (5)	104
C9—H9⋯O3	0.98	2.48	2.997 (4)	113
C11—H11*B*⋯O4	0.97	2.56	3.042 (5)	111
C14—H14⋯O1	0.93	2.45	2.808 (5)	103
C19—H19*C*⋯O7	0.96	2.41	3.065 (6)	125
C20—H20*B*⋯O5	0.96	2.33	2.939 (7)	121
C7—H7⋯*Cg*5^i^	0.98	2.85	3.724	148
C22—H22*A*⋯*Cg*6^ii^	0.97	2.97	3.646	128

Symmetry codes: (i) 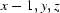; (ii) 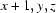.

**Fig. 1.**
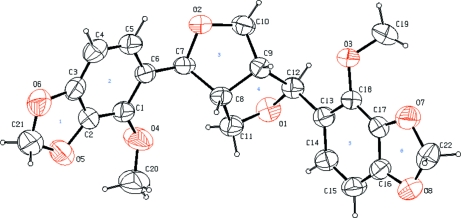
The asymmetric unit of (I) with the atoms labelled and displacement ellipsoids depicted at the 50% probability level for all non-H atoms. H-atoms are drawn as spheres of arbitrary radius.

## supplementary crystallographic information

### Comment

1,3-benzodioxole (methylenedioxyphenyl) moiety is frequently found in natural products, and has been reported to possess some interesting biological activities (Beroza & Barthel, 1957 ; Hu *et al.*,  2005 ; Hu *et al.*,  2007 ; Sonar *et al.*,  2006 ; Wang *et al.*,  2004 ; Yin *et al.*,  2007 ; Yu *et al.*,  2006 ; Zheng *et al.*, 2005*a*,b). The methylenedioxy positional isomers of oxolinic acid have been found to have widespread clinical applications and are used in the treatment of urinary tract infections (Mitscher *et al.*, 1979). They are also used for the synthesis of antimalarial drugs (Chien & Cheng, 1970). The title compound, (I), is obtained as part of our investigation on 1,3-benzodioxole derivatives.

In (I) (Fig. 1), both the 1,3-benzodioxole rings are almost planar. The planarity of the dioxole moiety is similar to those observed in related compounds (Zhou *et al.* 2007; Liang 2004, Zhang *et al.* 2007; Betz *et al.* 2007) and in contrast to the envelope conformation observed for these rings in few related compounds (Fu *et al.* 2006; Qi *et al.* 2006; Hussain *et al.* 2006). The *O*-methoxy group attached to each of the 1,3-benzodioxole rings differ in their orientation as shown by the corresponding torsion angles which probably explains the acentricity of the crystal. The tetrahydro furofuranyl ring adopts a distorted envelope-distorted envelope conformation as shown by the corresponding torsion angles (Table 1). The two five membered rings are fused in such a way that they share a common base described by the bond C8—C9 (Fig. 1). The Cremer & Pople (1975) puckering parameters for the O1—C11—C8—C9—C12 ring are Q(2) = 0.311 (3)Å and φ(2) = 163.0 (6)° whereas those for the O2—C7—C8—C9—C10 ring are Q(2) = 0.371 (3)Å and φ(2) = 162.9 (5)°. These values indicate that the extent of puckering is almost similar in both the rings. The pseudorotation parameters (Rao *et al.* 1981) for O1—C11—C8—C9—C12 ring are P = 254.8 (3)° & τ(*M*) = 34.7 (2)° for the C8—C9 reference bond with the closest pucker descriptor being twisted on C12—O1 and those for O2—C7—C8—C9—C10 ring are P = 254.1 (3)° and τ(*M*) = 41.4 (2)° for the C8—C9 reference bond with the closest puckering descriptor being twisted on C10—O2.

The crystal structure of (I) is stabilized by the interplay of intramolecular C—H···O, intermolecular C—H···π (Table 2) and π–π interactions (Fig. 2). The H-bond distances found in (I) agree with those reported in literature (Desiraju & Steiner, 1999; Desiraju, 1989). S(5) motifs (Bernstein *et al.*, 1995; Etter, 1990) are generated by each of C5—H5···O2 and C14—H14···O1 interactions. Each of the C9—H9···O3, C19—H19C···O7 and C20—H20B···O5 interactions generate an S(6) motif. An S(7) motif is generated by C11—H11B···O4 interaction. In Table 2, *Cg*3 refers to the centroid of the ring formed by O5, C2, C3, O6 & C21, *Cg*4 refers to the centroid of the ring formed by O7, C17, C16, O8 & C22, *Cg*5 refers to the centroid of the ring formed by C1—C6 and *Cg*6 refers to the centroid of the ring formed by C13—C18. A significant π–π stacking is observed between *Cg*4 and *Cg*6 (1 + *x*, *y*, *z*) with a centroid to centroid distance of 3.755 Å, a plane to plane distance of 3.633Å and an offset angle of 14.6°.

### Experimental

Ethanolic extract of powdered root of Ecbolium Viride (Forssk) spring was charged on a column and eluted with solvents ranging from non-polar to polar at the rate of 30 drops per minute. 34 fractions were collected, each of volume 25 ml with different ratios of solvents. The fractions collected were monitored by thin layer chromatography (TLC) for homogenity and similar fractions were pooled together. The title compound was isolated from one such pool. Diffraction quality crystals of the title compound were obtained by recrystallization from chloroform.

### Refinement

Hydrogen atoms were positioned geometrically (aromatic C—H = 0.93 Å, methine C—H = 0.98 Å, methylene C—H = 0.97Å & methyl C—H = 0.96 Å) and refined using a riding model. The hydrogen atom isotropic displacement parameters were fixed; *U*_iso_(aromatic H, methine H, methylene H) = 1.2 times *U*_eq_ of the parent atom; *U*_iso_(methyl H) = 1.5 times *U*_eq_ of the parent atom.

In the absence of significant anomalous scattering, the absolute configuration could not be reliably determined and then the Friedel pairs were merged and any references to the Flack parameter were removed.

### Crystal data

**Table:**

C_22_H_22_O_8_	*F*_000_ = 436
*M**_r_* = 414.40	*D*_x_ = 1.425 Mg m^−^^3^
Monoclinic, *P*2_1_	Mo *K*α radiation λ = 0.71073 Å
Hall symbol: P 2yb	Cell parameters from 25 reflections
*a* = 4.754 (5) Å	θ = 10–14º
*b* = 13.982 (4) Å	µ = 0.11 mm^−^^1^
*c* = 14.672 (5) Å	*T* = 293 (2) K
β = 97.97 (6)º	Prismatic, colorless
*V* = 965.8 (10) Å^3^	0.3 × 0.3 × 0.3 mm
*Z* = 2	

### Data collection

**Table:**

Enraf–Nonius CAD-4 diffractometer	*R*_int_ = 0.009
Radiation source: fine-focus sealed tube	θ_max_ = 25.0º
Monochromator: graphite	θ_min_ = 2.0º
*T* = 293(2) K	*h* = 0→5
ω–2θ scans	*k* = 0→16
Absorption correction: ψ scan(North *et al.*, 1968)	*l* = −17→17
*T*_min_ = 0.805, *T*_max_ = 0.999	2 standard reflections
2000 measured reflections	every 100 reflections
1777 independent reflections	intensity decay: none
1505 reflections with *I* > 2σ(*I*)	

### Refinement

**Table:**

Refinement on *F*^2^	Secondary atom site location: difference Fourier map
Least-squares matrix: full	Hydrogen site location: inferred from neighbouring sites
*R*[*F*^2^ > 2σ(*F*^2^)] = 0.040	H-atom parameters constrained
*wR*(*F*^2^) = 0.110	*w* = 1/[σ^2^(*F*_o_^2^) + (0.0741*P*)^2^ + 0.1102*P*] where *P* = (*F*_o_^2^ + 2*F*_c_^2^)/3
*S* = 1.05	(Δ/σ)_max_ < 0.001
1777 reflections	Δρ_max_ = 0.20 e Å^−^^3^
272 parameters	Δρ_min_ = −0.20 e Å^−^^3^
1 restraint	Extinction correction: SHELXL97 (Sheldrick, 2008), Fc^*^=kFc[1+0.001xFc^2^λ^3^/sin(2θ)]^-1/4^
Primary atom site location: structure-invariant direct methods	Extinction coefficient: 0.015 (4)

### Special details

**Table:**

Experimental. Psi-scan (North, *et al.*,1968). Number of psi-scan sets used was 3 Theta correction was applied. Averaged transmission function was used. No Fourier smoothing was applied.
Geometry. All e.s.d.'s (except the e.s.d. in the dihedral angle between two l.s. planes) are estimated using the full covariance matrix. The cell e.s.d.'s are taken into account individually in the estimation of e.s.d.'s in distances, angles and torsion angles; correlations between e.s.d.'s in cell parameters are only used when they are defined by crystal symmetry. An approximate (isotropic) treatment of cell e.s.d.'s is used for estimating e.s.d.'s involving l.s. planes.
Refinement. Refinement of *F*^2^ against ALL reflections. The weighted *R*-factor *wR* and goodness of fit *S* are based on *F*^2^, conventional *R*-factors *R* are based on *F*, with *F* set to zero for negative *F*^2^. The threshold expression of *F*^2^ > σ(*F*^2^) is used only for calculating *R*-factors(gt) *etc*. and is not relevant to the choice of reflections for refinement. *R*-factors based on *F*^2^ are statistically about twice as large as those based on *F*, and *R*- factors based on ALL data will be even larger.

### Fractional atomic coordinates and isotropic or equivalent isotropic displacement parameters (Å^2^)

**Table:**

	*x*	*y*	*z*	*U*_iso_*/*U*_eq_	
C1	1.0773 (8)	0.1633 (3)	1.4216 (2)	0.0526 (8)	
C2	1.2921 (8)	0.1468 (3)	1.4925 (2)	0.0561 (9)	
C3	1.4503 (8)	0.2198 (3)	1.5347 (2)	0.0570 (9)	
C4	1.4085 (9)	0.3134 (3)	1.5089 (3)	0.0639 (10)	
H4	1.5187	0.3627	1.5376	0.077*	
C5	1.1918 (8)	0.3303 (3)	1.4372 (2)	0.0573 (9)	
H5	1.1555	0.3931	1.4182	0.069*	
C6	1.0278 (7)	0.2588 (2)	1.3927 (2)	0.0453 (7)	
C7	0.7982 (7)	0.2786 (2)	1.3134 (2)	0.0442 (7)	
H7	0.6163	0.2557	1.3294	0.053*	
C8	0.8479 (6)	0.2337 (2)	1.2209 (2)	0.0427 (7)	
H8	1.0446	0.2118	1.2232	0.051*	
C9	0.7821 (6)	0.3148 (2)	1.1502 (2)	0.0447 (7)	
H9	0.9549	0.3388	1.1285	0.054*	
C10	0.6472 (8)	0.3900 (3)	1.2056 (2)	0.0538 (8)	
H10A	0.6794	0.4537	1.1829	0.065*	
H10B	0.4442	0.3795	1.2016	0.065*	
C11	0.6397 (8)	0.1550 (3)	1.1852 (2)	0.0539 (8)	
H11A	0.7398	0.1024	1.1609	0.065*	
H11B	0.5436	0.1308	1.2346	0.065*	
C12	0.5768 (6)	0.2704 (3)	1.0716 (2)	0.0464 (7)	
H12	0.4349	0.3185	1.0483	0.056*	
C13	0.7205 (6)	0.2343 (2)	0.9919 (2)	0.0456 (7)	
C14	0.7185 (8)	0.1376 (3)	0.9672 (2)	0.0536 (8)	
H14	0.6241	0.0944	1.0004	0.064*	
C15	0.8500 (8)	0.1038 (3)	0.8959 (3)	0.0589 (9)	
H15	0.8475	0.0391	0.8809	0.071*	
C16	0.9849 (7)	0.1695 (3)	0.8480 (2)	0.0517 (8)	
C17	0.9892 (7)	0.2650 (3)	0.8700 (2)	0.0488 (8)	
C18	0.8592 (8)	0.2999 (2)	0.9410 (2)	0.0477 (8)	
C19	0.7762 (12)	0.4613 (3)	0.8991 (3)	0.0808 (13)	
H19A	0.7930	0.5244	0.9249	0.121*	
H19B	0.5812	0.4488	0.8754	0.121*	
H19C	0.8900	0.4567	0.8500	0.121*	
C20	1.0043 (15)	0.0036 (3)	1.3671 (4)	0.1010 (18)	
H20A	0.8569	−0.0359	1.3354	0.151*	
H20B	1.0690	−0.0234	1.4264	0.151*	
H20C	1.1598	0.0070	1.3319	0.151*	
C21	1.6026 (13)	0.0854 (4)	1.6057 (3)	0.0889 (15)	
H21A	1.5541	0.0659	1.6650	0.107*	
H21B	1.7749	0.0522	1.5956	0.107*	
C22	1.2286 (9)	0.2467 (3)	0.7507 (3)	0.0673 (11)	
H22A	1.4341	0.2467	0.7551	0.081*	
H22B	1.1501	0.2621	0.6878	0.081*	
O1	0.4386 (5)	0.19560 (19)	1.11427 (16)	0.0549 (6)	
O2	0.7797 (5)	0.37966 (17)	1.29739 (16)	0.0551 (6)	
O3	0.8695 (7)	0.39473 (18)	0.96665 (16)	0.0666 (8)	
O4	0.8987 (7)	0.0950 (2)	1.3780 (2)	0.0811 (9)	
O5	1.3775 (8)	0.0610 (2)	1.5348 (2)	0.0890 (10)	
O6	1.6475 (7)	0.1843 (3)	1.60497 (19)	0.0784 (9)	
O7	1.1404 (7)	0.3157 (2)	0.81173 (18)	0.0717 (8)	
O8	1.1315 (7)	0.1546 (2)	0.77478 (18)	0.0698 (8)	

### Atomic displacement parameters (Å^2^)

**Table:**

	*U*^11^	*U*^22^	*U*^33^	*U*^12^	*U*^13^	*U*^23^
C1	0.066 (2)	0.0420 (19)	0.0494 (16)	−0.0059 (16)	0.0081 (15)	−0.0034 (15)
C2	0.072 (2)	0.049 (2)	0.0470 (16)	0.0097 (18)	0.0078 (16)	0.0039 (15)
C3	0.066 (2)	0.064 (2)	0.0413 (16)	0.0050 (18)	0.0093 (15)	−0.0087 (16)
C4	0.070 (2)	0.060 (2)	0.060 (2)	−0.0094 (19)	0.0025 (18)	−0.0160 (18)
C5	0.072 (2)	0.0424 (19)	0.0578 (19)	−0.0023 (18)	0.0101 (17)	−0.0048 (16)
C6	0.0534 (17)	0.0396 (18)	0.0451 (16)	−0.0021 (14)	0.0140 (13)	−0.0019 (14)
C7	0.0504 (16)	0.0331 (15)	0.0510 (17)	0.0020 (14)	0.0133 (14)	−0.0033 (12)
C8	0.0403 (15)	0.0387 (16)	0.0506 (17)	0.0012 (13)	0.0114 (12)	−0.0020 (13)
C9	0.0425 (15)	0.0436 (18)	0.0485 (16)	−0.0009 (14)	0.0076 (13)	0.0026 (14)
C10	0.0589 (19)	0.0419 (18)	0.0607 (19)	0.0068 (16)	0.0085 (15)	0.0016 (15)
C11	0.0611 (19)	0.042 (2)	0.0588 (19)	−0.0079 (15)	0.0077 (16)	−0.0042 (15)
C12	0.0387 (14)	0.0482 (18)	0.0520 (17)	0.0019 (14)	0.0055 (13)	−0.0007 (15)
C13	0.0396 (15)	0.0458 (18)	0.0492 (16)	0.0050 (14)	−0.0017 (12)	−0.0042 (14)
C14	0.0576 (19)	0.0450 (19)	0.0572 (18)	−0.0077 (16)	0.0045 (15)	−0.0040 (16)
C15	0.069 (2)	0.0413 (19)	0.065 (2)	0.0019 (17)	0.0010 (18)	−0.0112 (16)
C16	0.0542 (18)	0.052 (2)	0.0465 (16)	0.0090 (16)	−0.0004 (14)	−0.0085 (15)
C17	0.0527 (17)	0.0468 (19)	0.0455 (16)	0.0056 (15)	0.0018 (13)	0.0025 (14)
C18	0.0589 (18)	0.0386 (17)	0.0449 (16)	0.0080 (14)	0.0049 (14)	−0.0018 (13)
C19	0.119 (4)	0.043 (2)	0.078 (3)	0.010 (2)	0.006 (3)	0.008 (2)
C20	0.138 (5)	0.050 (3)	0.106 (4)	−0.008 (3)	−0.015 (4)	−0.011 (2)
C21	0.112 (4)	0.076 (3)	0.072 (3)	0.026 (3)	−0.012 (3)	−0.004 (2)
C22	0.066 (2)	0.084 (3)	0.054 (2)	0.002 (2)	0.0141 (17)	−0.010 (2)
O1	0.0417 (10)	0.0593 (16)	0.0635 (14)	−0.0085 (11)	0.0067 (10)	−0.0018 (12)
O2	0.0706 (15)	0.0394 (12)	0.0549 (13)	0.0086 (11)	0.0078 (12)	−0.0048 (10)
O3	0.109 (2)	0.0374 (13)	0.0530 (13)	0.0118 (14)	0.0112 (13)	−0.0009 (11)
O4	0.094 (2)	0.0412 (16)	0.098 (2)	−0.0100 (14)	−0.0195 (17)	0.0001 (14)
O5	0.127 (3)	0.0578 (18)	0.0730 (18)	0.0080 (18)	−0.0191 (18)	0.0092 (15)
O6	0.0845 (19)	0.086 (2)	0.0592 (15)	0.0131 (17)	−0.0101 (14)	−0.0071 (15)
O7	0.100 (2)	0.0573 (16)	0.0647 (15)	0.0025 (15)	0.0367 (15)	0.0019 (13)
O8	0.0881 (19)	0.0635 (18)	0.0603 (15)	0.0155 (15)	0.0191 (14)	−0.0102 (13)

### Geometric parameters (Å, °)

**Table:**

C1—C2	1.373 (5)	C12—H12	0.9800
C1—O4	1.375 (5)	C13—C14	1.399 (5)
C1—C6	1.411 (5)	C13—C18	1.403 (5)
C2—C3	1.364 (6)	C14—C15	1.374 (5)
C2—O5	1.385 (5)	C14—H14	0.9300
C3—C4	1.370 (6)	C15—C16	1.370 (6)
C3—O6	1.385 (5)	C15—H15	0.9300
C4—C5	1.387 (6)	C16—C17	1.372 (5)
C4—H4	0.9300	C16—O8	1.375 (4)
C5—C6	1.376 (5)	C17—C18	1.373 (5)
C5—H5	0.9300	C17—O7	1.386 (5)
C6—C7	1.506 (5)	C18—O3	1.377 (4)
C7—O2	1.433 (4)	C19—O3	1.387 (5)
C7—C8	1.544 (4)	C19—H19A	0.9600
C7—H7	0.9800	C19—H19B	0.9600
C8—C11	1.524 (5)	C19—H19C	0.9600
C8—C9	1.539 (4)	C20—O4	1.390 (6)
C8—H8	0.9800	C20—H20A	0.9600
C9—C10	1.525 (5)	C20—H20B	0.9600
C9—C12	1.535 (5)	C20—H20C	0.9600
C9—H9	0.9800	C21—O6	1.400 (6)
C10—O2	1.412 (4)	C21—O5	1.426 (6)
C10—H10A	0.9700	C21—H21A	0.9700
C10—H10B	0.9700	C21—H21B	0.9700
C11—O1	1.430 (5)	C22—O7	1.419 (5)
C11—H11A	0.9700	C22—O8	1.428 (6)
C11—H11B	0.9700	C22—H22A	0.9700
C12—O1	1.425 (4)	C22—H22B	0.9700
C12—C13	1.520 (4)		
			
C2—C1—O4	125.6 (3)	C13—C12—H12	108.7
C2—C1—C6	117.5 (3)	C9—C12—H12	108.7
O4—C1—C6	116.8 (3)	C14—C13—C18	118.7 (3)
C3—C2—C1	121.6 (3)	C14—C13—C12	122.2 (3)
C3—C2—O5	109.6 (3)	C18—C13—C12	119.1 (3)
C1—C2—O5	128.8 (4)	C15—C14—C13	122.8 (3)
C2—C3—C4	122.7 (3)	C15—C14—H14	118.6
C2—C3—O6	110.1 (4)	C13—C14—H14	118.6
C4—C3—O6	127.2 (4)	C16—C15—C14	117.0 (3)
C3—C4—C5	115.9 (4)	C16—C15—H15	121.5
C3—C4—H4	122.1	C14—C15—H15	121.5
C5—C4—H4	122.1	C15—C16—C17	121.6 (3)
C6—C5—C4	123.3 (4)	C15—C16—O8	128.6 (3)
C6—C5—H5	118.4	C17—C16—O8	109.8 (3)
C4—C5—H5	118.4	C16—C17—C18	122.1 (3)
C5—C6—C1	119.1 (3)	C16—C17—O7	110.1 (3)
C5—C6—C7	122.3 (3)	C18—C17—O7	127.8 (3)
C1—C6—C7	118.6 (3)	C17—C18—O3	123.2 (3)
O2—C7—C6	109.3 (3)	C17—C18—C13	117.7 (3)
O2—C7—C8	105.6 (3)	O3—C18—C13	119.1 (3)
C6—C7—C8	114.9 (3)	O3—C19—H19A	109.5
O2—C7—H7	109.0	O3—C19—H19B	109.5
C6—C7—H7	109.0	H19A—C19—H19B	109.5
C8—C7—H7	109.0	O3—C19—H19C	109.5
C11—C8—C9	103.8 (3)	H19A—C19—H19C	109.5
C11—C8—C7	115.1 (3)	H19B—C19—H19C	109.5
C9—C8—C7	104.6 (2)	O4—C20—H20A	109.5
C11—C8—H8	111.0	O4—C20—H20B	109.5
C9—C8—H8	111.0	H20A—C20—H20B	109.5
C7—C8—H8	111.0	O4—C20—H20C	109.5
C10—C9—C12	114.1 (3)	H20A—C20—H20C	109.5
C10—C9—C8	102.1 (2)	H20B—C20—H20C	109.5
C12—C9—C8	104.9 (3)	O6—C21—O5	109.3 (4)
C10—C9—H9	111.7	O6—C21—H21A	109.8
C12—C9—H9	111.7	O5—C21—H21A	109.8
C8—C9—H9	111.7	O6—C21—H21B	109.8
O2—C10—C9	105.8 (3)	O5—C21—H21B	109.8
O2—C10—H10A	110.6	H21A—C21—H21B	108.3
C9—C10—H10A	110.6	O7—C22—O8	108.8 (3)
O2—C10—H10B	110.6	O7—C22—H22A	109.9
C9—C10—H10B	110.6	O8—C22—H22A	109.9
H10A—C10—H10B	108.7	O7—C22—H22B	109.9
O1—C11—C8	107.4 (3)	O8—C22—H22B	109.9
O1—C11—H11A	110.2	H22A—C22—H22B	108.3
C8—C11—H11A	110.2	C12—O1—C11	108.0 (2)
O1—C11—H11B	110.2	C10—O2—C7	105.5 (3)
C8—C11—H11B	110.2	C18—O3—C19	117.0 (3)
H11A—C11—H11B	108.5	C1—O4—C20	118.8 (4)
O1—C12—C13	112.2 (3)	C2—O5—C21	105.2 (4)
O1—C12—C9	104.5 (2)	C3—O6—C21	105.7 (3)
C13—C12—C9	113.9 (3)	C17—O7—C22	105.4 (3)
O1—C12—H12	108.7	C16—O8—C22	105.8 (3)
			
O4—C1—C2—C3	−176.8 (4)	C9—C12—C13—C18	−63.3 (4)
C6—C1—C2—C3	0.9 (5)	C18—C13—C14—C15	0.9 (5)
O4—C1—C2—O5	0.5 (6)	C12—C13—C14—C15	−179.3 (3)
C6—C1—C2—O5	178.1 (4)	C13—C14—C15—C16	−0.5 (5)
C1—C2—C3—C4	−0.7 (6)	C14—C15—C16—C17	0.1 (5)
O5—C2—C3—C4	−178.5 (4)	C14—C15—C16—O8	179.8 (3)
C1—C2—C3—O6	178.7 (3)	C15—C16—C17—C18	0.0 (5)
O5—C2—C3—O6	0.9 (4)	O8—C16—C17—C18	−179.8 (3)
C2—C3—C4—C5	0.7 (6)	C15—C16—C17—O7	179.6 (3)
O6—C3—C4—C5	−178.6 (3)	O8—C16—C17—O7	−0.2 (4)
C3—C4—C5—C6	−0.8 (6)	C16—C17—C18—O3	177.5 (3)
C4—C5—C6—C1	1.0 (5)	O7—C17—C18—O3	−2.0 (5)
C4—C5—C6—C7	−178.5 (3)	C16—C17—C18—C13	0.4 (5)
C2—C1—C6—C5	−1.0 (5)	O7—C17—C18—C13	−179.1 (3)
O4—C1—C6—C5	176.9 (3)	C14—C13—C18—C17	−0.8 (4)
C2—C1—C6—C7	178.5 (3)	C12—C13—C18—C17	179.4 (3)
O4—C1—C6—C7	−3.6 (5)	C14—C13—C18—O3	−178.0 (3)
C5—C6—C7—O2	−2.0 (4)	C12—C13—C18—O3	2.2 (4)
C1—C6—C7—O2	178.5 (3)	C13—C12—O1—C11	89.0 (3)
C5—C6—C7—C8	116.5 (3)	C9—C12—O1—C11	−34.9 (3)
C1—C6—C7—C8	−63.0 (4)	C8—C11—O1—C12	29.6 (3)
O2—C7—C8—C11	−126.6 (3)	C9—C10—O2—C7	−42.3 (3)
C6—C7—C8—C11	112.9 (3)	C6—C7—O2—C10	158.5 (3)
O2—C7—C8—C9	−13.3 (3)	C8—C7—O2—C10	34.3 (3)
C6—C7—C8—C9	−133.8 (3)	C17—C18—O3—C19	55.8 (5)
C11—C8—C9—C10	110.4 (3)	C13—C18—O3—C19	−127.2 (4)
C7—C8—C9—C10	−10.7 (3)	C2—C1—O4—C20	−34.0 (6)
C11—C8—C9—C12	−9.0 (3)	C6—C1—O4—C20	148.4 (4)
C7—C8—C9—C12	−130.0 (3)	C3—C2—O5—C21	−0.7 (5)
C12—C9—C10—O2	144.6 (3)	C1—C2—O5—C21	−178.2 (4)
C8—C9—C10—O2	32.0 (3)	O6—C21—O5—C2	0.2 (6)
C9—C8—C11—O1	−11.5 (3)	C2—C3—O6—C21	−0.8 (5)
C7—C8—C11—O1	102.2 (3)	C4—C3—O6—C21	178.6 (5)
C10—C9—C12—O1	−84.5 (3)	O5—C21—O6—C3	0.3 (6)
C8—C9—C12—O1	26.4 (3)	C16—C17—O7—C22	0.7 (4)
C10—C9—C12—C13	152.6 (3)	C18—C17—O7—C22	−179.7 (3)
C8—C9—C12—C13	−96.4 (3)	O8—C22—O7—C17	−1.0 (4)
O1—C12—C13—C14	−1.6 (4)	C15—C16—O8—C22	179.8 (4)
C9—C12—C13—C14	116.9 (4)	C17—C16—O8—C22	−0.4 (4)
O1—C12—C13—C18	178.2 (3)	O7—C22—O8—C16	0.9 (4)

### Hydrogen-bond geometry (Å, °)

**Table:**

*D*—H···*A*	*D*—H	H···*A*	*D*···*A*	*D*—H···*A*
C5—H5···O2	0.93	2.34	2.721 (5)	104
C9—H9···O3	0.98	2.48	2.997 (4)	113
C11—H11B···O4	0.97	2.56	3.042 (5)	111
C14—H14···O1	0.93	2.45	2.808 (5)	103
C19—H19C···O7	0.96	2.41	3.065 (6)	125
C20—H20B···O5	0.96	2.33	2.939 (7)	121
C7—H7···Cg5^i^	0.98	2.86	3.724	148
C22—H22A···Cg6^ii^	0.97	2.97	3.646	128

Symmetry codes: (i) *x*−1, *y*, *z*; (ii) *x*+1, *y*, *z*.
